# Pericardial cyst in a one-year-old boy with ventricular septal defect and patent ductus arteriosus

**DOI:** 10.1186/s43044-022-00278-6

**Published:** 2022-05-16

**Authors:** Nirmal Panthee, Sidhartha Pradhan, Raamesh Koirala, Anil Dev Pant, Bishow Pokhrel, Subhash Chandra Shah, Rabindra Bhakta Timala

**Affiliations:** 1 Department of Cardiac Surgery, Shahid Gangalal National Heart Center, Bansbari, Kathmandu, Nepal; 2 Department of Pathology, Tribhuvan University Teaching Hospital, Maharajgunj, Kathmandu, Nepal; 3 Department of Pediatric Cardiology, Shahid Gangalal National Heart Center, Bansbari, Kathmandu, Nepal

**Keywords:** Pericardial cyst, Congenital heart disease, Ventricular septal defect, Patent ductus arteriosus

## Abstract

**Background:**

Pericardial cysts are rare, with the most common etiology being congenital. Ventricular septal defect is the most common congenital heart disease in children. However, the combination of pericardial cyst, ventricular septal defect, and patent ductus arteriosus is extremely rare.

**Case presentation:**

A one-year-old boy with ventricular septal defect and patent ductus arteriosus was planned for surgical correction. Intraoperatively, we made an additional diagnosis of a large pericardial cyst; and the cyst was excised along with ventricular septal defect closure and patent ductus arteriosus ligation.

**Conclusions:**

Pericardial cysts can sometimes be missed with transthoracic echocardiography. Excision of the cyst can safely be done during concomitant cardiac surgery.

**Supplementary Information:**

The online version contains supplementary material available at 10.1186/s43044-022-00278-6.

## Background

Pericardial cysts are rare, with incidence of 1:100,000 [1, 2]. Ventricular septal defects (VSD) have an incidence of 1.56–53.2 per 1000 live births; the detection being dramatically increased due to the use of advanced imaging techniques [3]. Patent ductus arteriosus (PDA) has an incidence of 1 per 2000 term infants [4]. Thus, the combination of pericardial cyst, VSD and PDA is very rare. We have previously reported an adult case of pericardial cyst with atrial septal defect [2]. Here, we report a case of one-year-old boy with pericardial cyst, VSD, and PDA who underwent VSD closure, PDA ligation, and excision of the pericardial cyst.

## Case presentation

A one-year-old boy weighing 10 kg was brought to us with repeated lower respiratory tract infections since birth. On examination, he was a playful boy with stable vital signs. Systemic oxygen saturation (SpO2) was 96% in room air. He did not have cyanosis, clubbing, edema, pallor, icterus, or jugular venous distension. Respiratory system examination was unremarkable. Cardiovascular examination revealed normal first and loud second heart sounds with systolic murmur in left lower sternal border.

Baseline hematologic and biochemical profiles were within normal limits. Electrocardiogram showed sinus rhythm with heart rate of 113 beats/min. Chest X-ray showed cardiothoracic ratio (CTR) of 0.55 with plethoric lung fields. Transthoracic echocardiography revealed dilated left ventricle (3.6/2.1 cm) with ejection fraction (EF) of 60%, anterior malaligned perimembranous VSD measuring 1 cm with left to right shunt, 3 mm PDA with left to right shunt, and severe pulmonary artery hypertension (tricuspid regurgitation pressure gradient (TRPG): 50 mmHg). With this diagnosis, VSD closure and PDA ligation was planned.

**Fig. 1:**
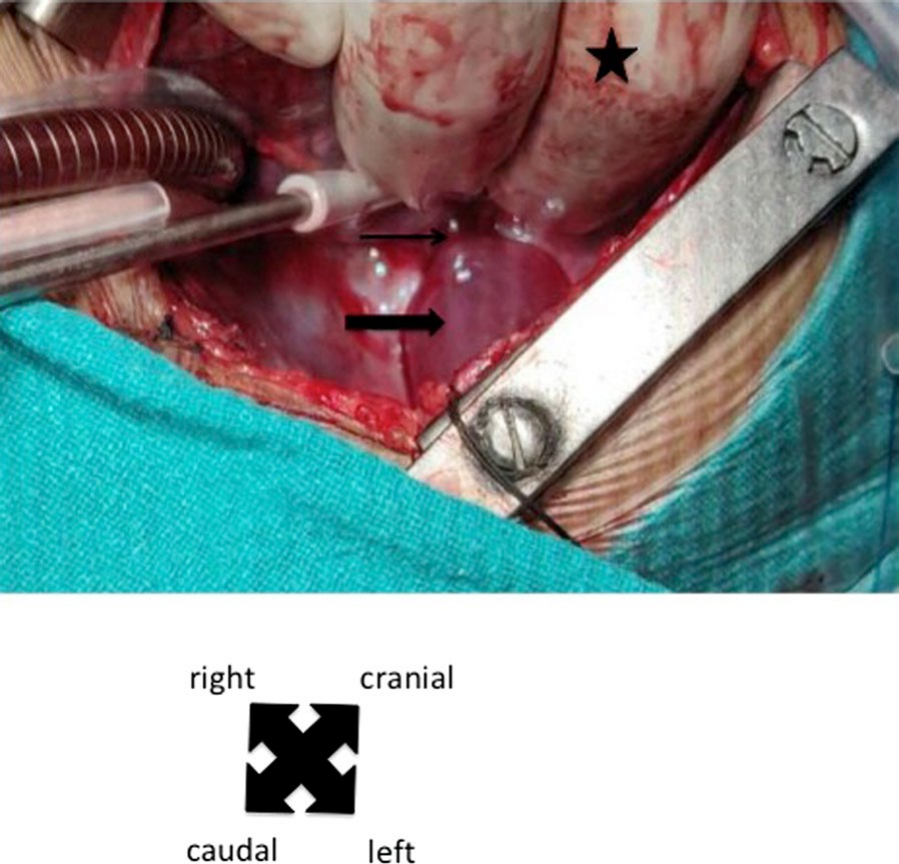
Cyst seen after lifting the apex of the heart. (thick arrow: cyst, thin arrow: stalk of the cyst attached to the pericardium near atrioventricular groove towards the left side, star: surgeon’s hand lifting the apex of the heart cranially to have a better view of the cyst

**Fig. 2:**
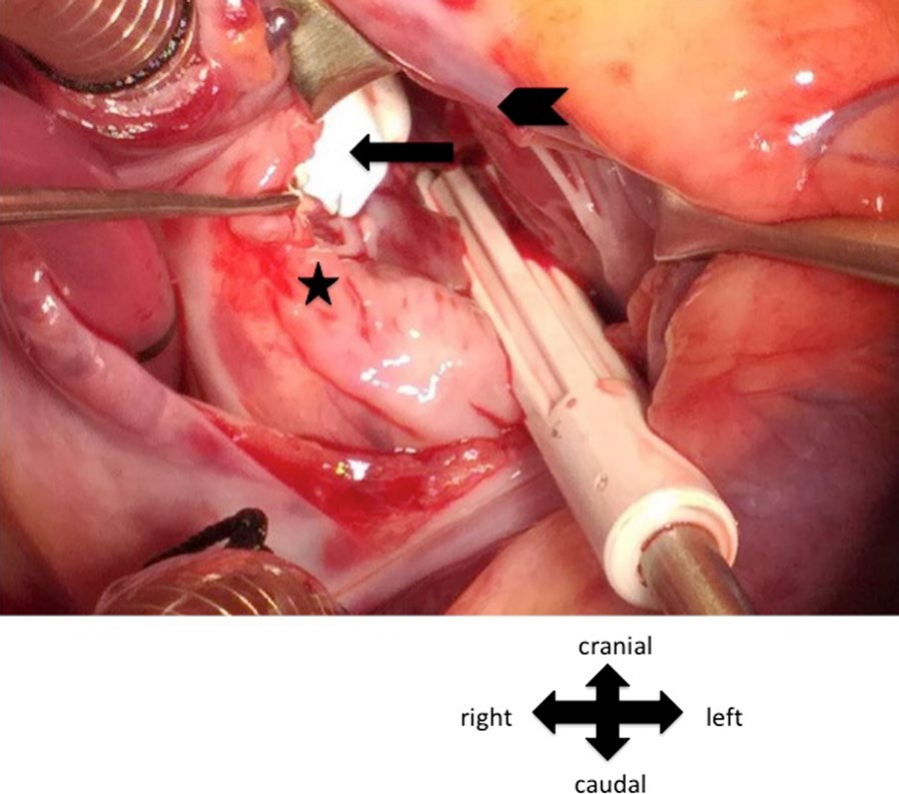
VSD was closed with ePTFE patch (arrow). Arrowhead: retracted cut margin of right atrium, star: septal leaflet of tricuspid valve

**Fig. 3:**
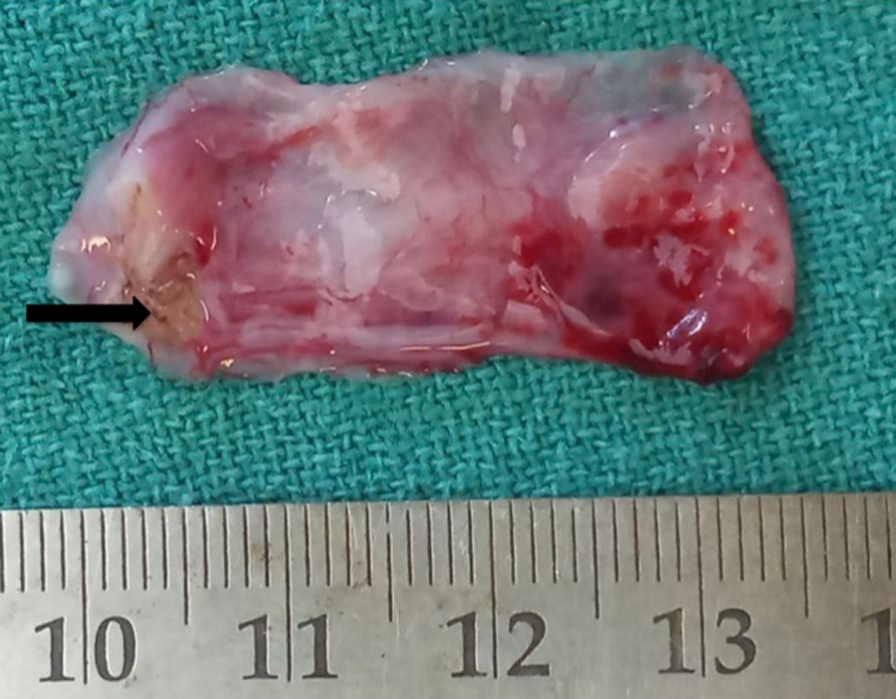
Excised specimen of the cyst. Arrow shows the stalk which was excised using electrocautery

**Fig. 4:**
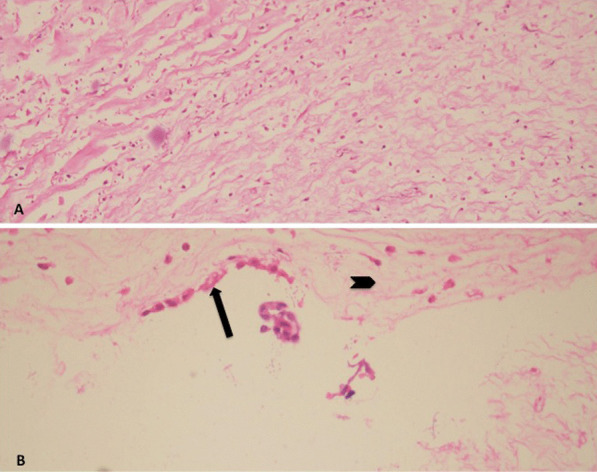
Histopathology of the resected cyst using Hematoxylin and Eosin (H&E) stain. **A** H&Ex20 shows fibro-collagenous wall with chronic inflammation (lymphocytes). **B** H&Ex40 shows mesothelial lining (cuboidal cells) of the cyst (arrow); and stroma (arrowhead)

Under general anesthesia, following median sternotomy and pericardiotomy, to our surprise, we noticed a freely mobile cystic lesion (3 × 4 cm) in the inferior surface of heart, which was attached by a stalk to the posterior pericardium near the atrioventricular groove on the left side. (Fig. 1, Additional file 1: video 1 and 2). Cardiopulmonary bypass (CPB) was established with ascending aortic and bicaval cannulation. After initiation of cardiopulmonary bypass, PDA was ligated with number 2 silk suture. After aortic cross clamp, cardiac arrest was achieved by antegrade cardioplegia. Right atrium was opened and VSD was closed with expanded polytetrafluoroethylene (ePTFE) patch using 6–0 prolene suture (Fig. 2). Right atrium was closed and aortic cross clamp was removed. Before weaning from the cardiopulmonary bypass, the apex of the heart was lifted up and the cyst was excised using electrocautery. The cyst contained clear fluid and the resected cyst is shown in Fig. 3. Total CPB time was 117 min and aortic cross clamp time was 80 min. After excision of the cyst, CBP was weaned off. The patient was transferred to pediatric intensive care unit with minimal inotropic support. He was extubated on the same day; transferred to general ward on 2^nd^ postoperative day; and discharged home on 5^th^ postoperative day. The histopathology of the resected specimen showed fibro-collagenous wall with mild inflammation and mesothelial lining consistent with pericardial cyst (Fig. 4).

## Discussion

Pericardial cysts are rare, with most common etiology being congenital [5]. Most pericardial cysts are asymptomatic and diagnosed incidentally, although hemorrhage into the cyst leading to chest pain, and cardiac tamponade have been reported [1]. Most of the available literature is in the form of case or case series report in asymptomatic adult patients. Reports of pericardial cysts in children have been scarce [6]. As of 2003, fewer than 20 cases of pericardial cysts were reported in the worldwide literature in children less than 18 years of age [7]. To our best knowledge, the youngest report was that of a 2-year-old boy [8]. Most of the pericardial cysts reported in pediatric age group (2-year-old boy, 3-year-old girl, 10-year-old girl) are from Japan [1, 8, 9]. The oldest case of pericardial cyst reported in the literature is of a 102-year-old woman from Cyprus [10]**.** We believe ours is one of the rarest reports of a one-year-old child with associated VSD and PDA.

Most of the cysts are diagnosed by transthoracic echocardiography; however, in our case, we missed to detect during preoperative echocardiography. Echocardiography is operator-dependent; and most of the times, we look for intracardiac lesions; and extracardiac lesions could be overlooked. Also, chest X-ray would show cardiomegaly or abnormal shaped cardiac silhouette; [9] however, in our case, the primary diagnosis of VSD and PDA would itself result into cardiomegaly, and due to location of the cyst (immediately behind the heart), abnormal silhouette was not expected. Computed tomography (CT) scan would definitely have detected the cyst. However, we do not routinely perform CT scan for patients with isolated VSD unless the VSD is part of tetralogy of Fallot.

Immediately after opening the chest, we were not sure about the content of the cyst. Keeping in mind the rare possibility of hydatid cyst, we deferred to excise the cyst until after the intracardiac procedure was completed and cardiac chambers were closed to avoid spillage of the cyst contents inside the heart itself. We do not believe that the management would have been significantly different from ours in any other cardiac center. We also do not believe that we have done a marvellous job by resecting a pericardial cyst in a child with VSD and PDA. However, we do believe that reporting our case would definitely add to the available literature given that this is a rare entity.

## Conclusions

We encountered pericardial cyst as an incidental finding during cardiac surgery and we have successfully resected a pericardial cyst as a concomitant operation during VSD closure and PDA ligation in a very young child.

## Supplementary Information

**Additional file 1:** Video 1. The cyst is seen freely mobile, which appears and disappears with cardiac contractility. Pump sucker tip is used to bring the cyst to our field of vision.

**Additional file 2:** Video 2. Cyst is seen all the time unlike in supplementary video 1.

## Data Availability

Upon reasonable request, the corresponding author will share the data and materials used in the manuscript.

## Abbreviations

CPB: Cardiopulmonary bypass
CT: Computed tomography
CTR: Cardiothoracic ratio
EF: Ejection fraction
ePTFE: Expanded polytetrafluoroethylene
H&E: Hematoxylin and Eosin
PDA: Patent ductus arteriosus
TRPG: Tricuspid regurgitation pressure gradient
VSD: Ventricular septal defect
